# Mutant KLF1 in Adult Anemic *Nan* Mice Leads to Profound Transcriptome Changes and Disordered Erythropoiesis

**DOI:** 10.1038/s41598-018-30839-2

**Published:** 2018-08-24

**Authors:** Danitza Nébor, Joel H. Graber, Steven L. Ciciotte, Raymond F. Robledo, Julien Papoin, Emily Hartman, Kevin R. Gillinder, Andrew C. Perkins, James J. Bieker, Lionel Blanc, Luanne L. Peters

**Affiliations:** 1 The Jackson Laboratory, Bar Harbor, ME 04609 USA; 2 MDI Biological Laboratory, Salisbury Cove, ME 04672 USA; 3 Feinstein Institute for Medical Research, Manhasset, NY 11030 USA; 4 Australian Centre for Blood Diseases, Monash University, Melbourne, VIC 3004 Australia; 5 The Alfred Hospital, Melbourne, VIC 3004 Australia; 6 Department of Cell, Developmental and Regenerative Biology, Mount Sinai School of Medicine, New York, NY 10029 USA

## Abstract

Anemic *Nan* mice carry a mutation (E339D) in the second zinc finger of erythroid transcription factor KLF1. Nan-KLF1 fails to bind a subset of normal KLF1 targets and ectopically binds a large set of genes not normally engaged by KLF1, resulting in a corrupted fetal liver transcriptome. Here, we performed RNAseq using flow cytometric-sorted spleen erythroid precursors from adult *Nan* and WT littermates rendered anemic by phlebotomy to identify global transcriptome changes specific to the *Nan Klf1* mutation as opposed to anemia generally. Mutant Nan-KLF1 leads to extensive and progressive transcriptome corruption in adult spleen erythroid precursors such that stress erythropoiesis is severely compromised. Terminal erythroid differentiation is defective in the bone marrow as well. Principle component analysis reveals two major patterns of differential gene expression predicting that defects in basic cellular processes including translation, cell cycle, and DNA repair could contribute to disordered erythropoiesis and anemia in *Nan*. Significant erythroid precursor stage specific changes were identified in some of these processes in *Nan*. Remarkably, however, despite expression changes in large numbers of associated genes, most basic cellular processes were intact in *Nan* indicating that developing red cells display significant physiological resiliency and establish new homeostatic set points *in vivo*.

## Introduction

In the inbred semi-dominant mouse model, *Nan* (neonatal anemia), heterozygotes suffer lifelong anemia due to a missense mutation (E339D) in KLF1 (Krüppel-like factor 1)^1,2^. Homozygotes die *in utero* (E10-11). *Klf1* expression is restricted to megakaryocyte-erythroid progenitors and erythroid lineage cells where it plays a global role in lineage determination and initiating and maintaining the erythroid-specific transcriptome including expression of globin, membrane skeleton, heme biosynthetic, iron regulating, and cell cycle genes^3–10^. Indeed, characteristics of both hereditary spherocytosis and thalassemia are evident in adult *Nan*/+ (hereafter *Nan*) mice with membrane skeleton defects, decreased expression of adult globin, and increased expression of embryonic globin^1,2^.

KLF1 is a C2H2 transcription factor with three tandem zinc fingers (ZFs), each recognizing a nucleotide triplet in its 9 bp cognate DNA binding site, 5′-CCM-CRC-CCN^3,11,12^. Glutamic acid at position 339 within ZF2 interacts with the middle triplet. Despite the conservative amino acid change to aspartic acid, the effect on DNA recognition is profound. Previous work^1^ showed that Nan-KLF1 binds normally to target genes containing guanine at position 5 in the middle triplet but fails to bind, or binds with reduced affinity, to genes containing adenine including β-globin (*Hbb*), the cell cycle regulator E2F2 (*E*2*f2*), and the membrane skeleton protein dematin (*Dmtn*). Notably, the normal *Klf1* allele is expressed in *Nan* erythroid cells. Despite this, expression of genes that mutant Nan-KLF1 does not bind is somehow disrupted through an unknown mechanism.

Nan-KLF1 also ectopically binds sites not normally engaged by KLF1^13,14^. In fetal liver, only 18% of up-regulated and 52% of down-regulated genes in *Nan* overlapped known KLF1 targets. ChIPseq confirmed ectopic Nan-KLF1 binding to an altered consensus sequence, CCM-NGC-CCN, with the result that >60% of Nan-KLF1 occupied sites do not overlap wild type (WT) KLF1 sites. Ectopic binding contributes to anemia in *Nan* through extrinsic mechanisms^15^. For example, hepcidin (*Hamp*) and interferon regulatory factor 7 (*Irf7*), normally expressed in adult liver^16^ and macrophages^17^, respectively, show dramatically increased expression in *Nan* fetal liver^13^ and adult WT spleen and bone marrow^15^ leading to increased serum hepcidin and interferon beta. lncreased hepcidin with markedly decreased erythroferrone, which is not bound by Nan-KLF1^15^, limits iron availability^18,19^. Interferons inhibit erythropoiesis at the BFU- and CFU-E stages^20^.

*Nan* is relevant to human erythroid-related disease. Human KLF1 mutations lead to benign defects^21,22^ and anemia, sometimes severe^23^. For example, a different substitution (E325K) at the position corresponding to *Nan* causes congenital dyserythropoietic anemia (CDA) type IV^22^. Prior transcriptome studies in *Nan* focused on fetal liver erythroid cells^1,13,15^. Here, we performed RNAseq in adult spleen erythroid precursors, comparing *Nan* to littermates made anemic by phlebotomy (WT-PHB). The use of anemic WT controls allowed identification of expression changes occurring primarily in response to the *Nan* KLF1 defect. We show that differential expression in *Nan* differs in fetal liver and spleen erythroid cells, that expression variation is driven primarily by precursor cell type with mutation effects most prominent in late erythroblasts, and that progression of erythropoiesis is significantly impaired. PCA reveals two major patterns of differential gene expression and predicts that defects in basic cellular processes (e.g., translation, cell cycle) contribute to anemia in *Nan*. Indeed, erythroid precursor stage specific alterations in some of these processes were identified. Overall, however, despite extensive differential expression, the basic cellular machinery appears largely intact in *Nan*, suggesting substantial tolerance to transcriptional changes and physiological resiliency in developing red blood cells (RBCs) *in vivo*.

## Results

### Distortion of the *Nan* transcriptome increases during differentiation

**Figure 1:**
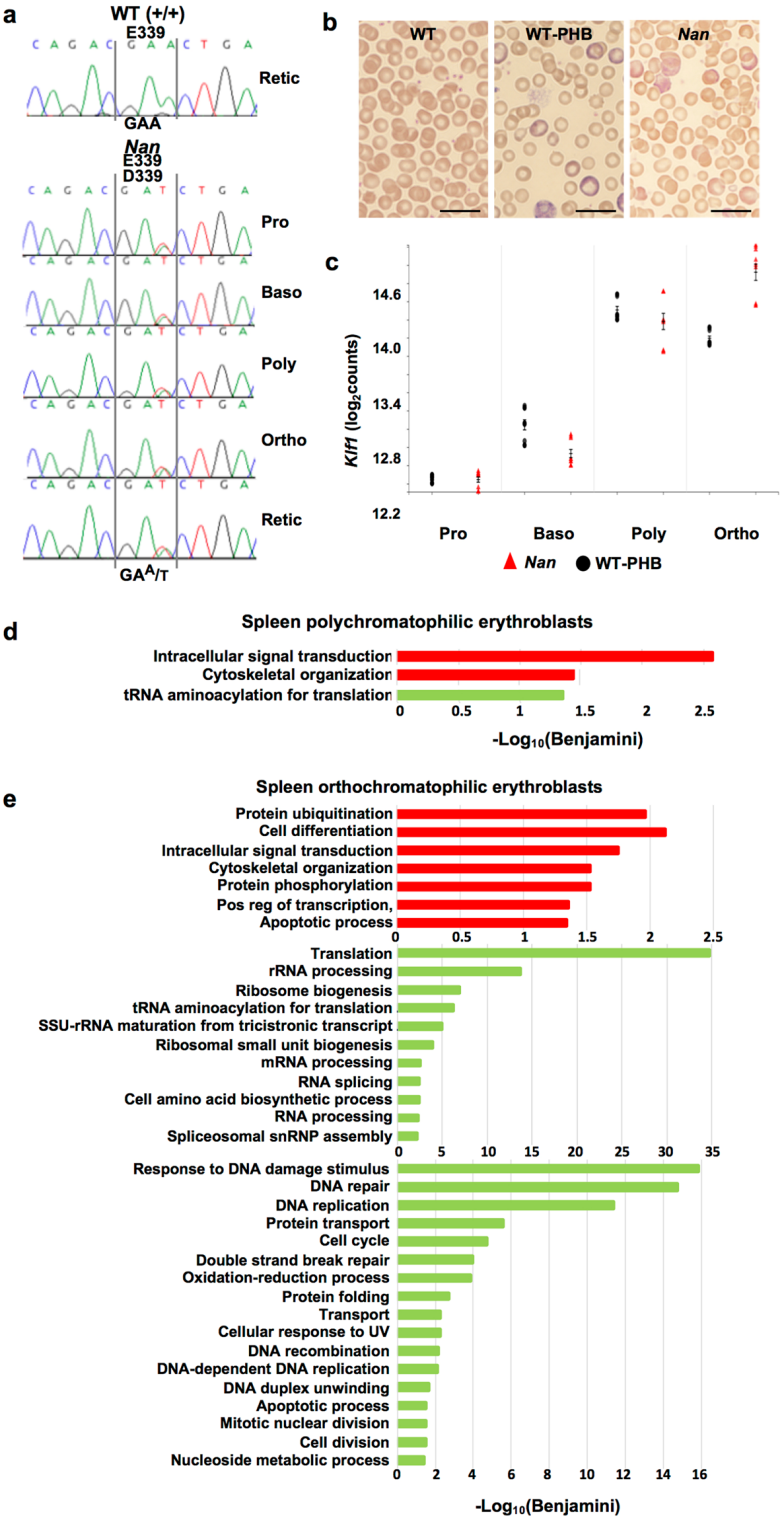
Aberrant erythroid transcriptome in adult anemic *Nan* mice. (**a**) cDNA sequence chromatograms showing transcription of *Klf1* alleles in wild type control (WT, +/+) and sorted *Nan* spleen erythroid cells. Both the normal and mutant alleles are equally expressed in *Nan* throughout terminal differentiation. (**b**) Peripheral blood smears from untreated (non-anemic) WT and phlebotomized WT control (WT-PHB) and *Nan* adult mice. Bars, 10 µM. (**c**) Expression level (log_2_ counts) of *Klf1* in WT-PHB and *Nan* spleen erythroid precursors. Differences in *Klf1* expression do not meet filtering criterion (fold change ≥2, false discovery rate ≤0.05) for differentially expressed genes in any of the precursor populations. The graph shows the three technical replicates for each of the 3 biological replicates, giving 9 points per genotype per cell type. Tick marks, mean ± SD. (**d**,**e**) Top functional annotations associated with up- (red) and down- (green) regulated gene expression in *Nan* spleen poly- and orthochromatophilic erythroblasts suggest multiple mechanisms could contribute to anemia in *Nan*. Note: In panel e terms broadly associated with translation and RNA processing are shown separately (middle) in order to display other highly significant functional annotations (bottom).

Both *Klf1* alleles, E339 and D339, are expressed in *Nan* spleen erythroid precursors (Fig. 1a), as previously demonstrated in fetal liver^1^. We performed RNAseq analysis on sorted erythroid precursors (pro-, basophilic, poly-, and orthochromatophilic erythroblasts) from adult male *Nan* and WT-PHB spleens to identify global transcriptome changes specific to Nan-KLF1. Peripheral blood studies confirm that WT-PHB mice develop anemia with significantly decreased RBC count, hemoglobin, and hematocrit; and increased RBC and hemoglobin distribution width, reticulocyte percentage, and spleen weight (Supplementary Table S1). Cell morphology is similar in *Nan* and WT-PHB with significant anisocytosis and polychromasia (Fig. 1b). Poikilocytosis including fragmented cells is rare. Circulating erythroblasts are not seen. Total bilirubin and LDH, indicators of hemolysis, did not differ significantly between *Nan* and WT-PHB (Supplementary Table S1); bilirubin, but not LDH, was increased over WT in both.

We obtained ~36 million mapped reads per sample (3 male mice per genotype with 4 erythroid precursor populations each for a total of 24 samples with 3 technical replicates each) and analyzed for differential expression. The number of differentially expressed genes (DEGs) increases dramatically during differentiation (Table 1), likely reflecting progressively increasing *Klf1* expression (Fig. 1c; expression profiles can be generated at http://nanexpression.mdibl.org/). In total, 6,965 unique genes were differentially expressed across the combined precursor populations. Surprisingly, these overlapped with only 61% of fetal liver DEGs. Analysis of gene ontology (GO) terms using DAVID^24^ by precursor cell type revealed no significant functional annotations (Benjamini corrected p-values) associated with pro- and basophilic erythroblasts and just three associated with polychromatophilic erythroblasts. A great many, however, emerge in orthochromatophilic erythroblasts including processes that could potentially contribute to anemia (translation, RNA processing, cytoskeletal organization, apoptosis, oxidation-reduction, cell cycle) (Fig. 1d,e). Indeed, cytoskeletal (membrane skeleton) defects were documented previously in *Nan*^1,2^. The most significant *p-*values are associated with down-regulated genes in orthochromatophilic erythroblasts, suggesting that failure to engage normal DNA targets, or engaging with reduced affinity, is a major consequence of Nan-KLF1 late in differentiation. The complete lists of DEGs in fetal liver, DEGs common to fetal liver and spleen, and DEGS in each of the four spleen precursor cell types are given in Supplementary Tables S2–S7.

**Table 1:** Differentially expressed genes in *Nan* spleen erythroid precursors*.

	Pro	Baso	Poly	Ortho
DEGs	1040	1659	2679	5125
Up-regulated	505	860	1438	2209
Down-regulated	535	799	1241	2916

*FC ≥ 2, FDR = 0.05.

### *Nan*-specific expression differences in fetal liver and spleen

**Figure 2:**
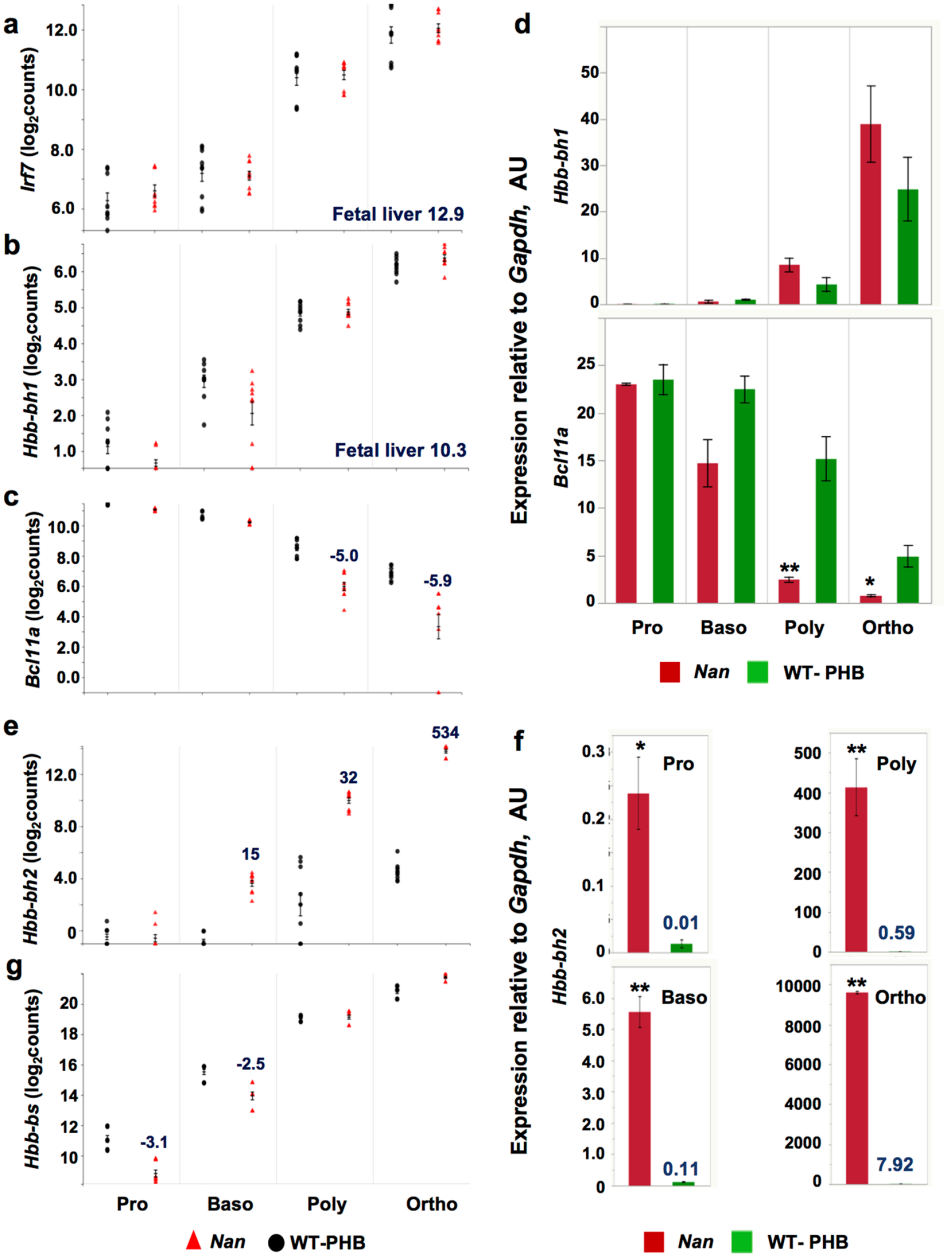
*Nan*- and erythroid niche-specific expression changes. Expression of (**a**) *Irf7* and (**b**) *Hbb-bh1* is not differentially regulated in *Nan* vs. WT-PHB spleen erythroid precursor cells while both are markedly upregulated in *Nan* fetal liver (fold change in *Nan* fetal liver from Supplemental Table 2 in blue). (**c**) Expression of *Bcl11a*. Fold change vs. WT-PHB in blue. (**d**) Confirmation of *Hbb-bh1* (top) and *Bcl11a* (bottom) expression by quantitative RT-PCR. AU, arbitrary units, no significant differences. n = 3 for *Nan* and WT-PHB. (**e**) *Hbb-bh2* is progressively upregulated during erythropoiesis in *Nan*, reaching a 534-fold increase at the orthochromatophilic stage. (**f**) Confirmation of dramatically up-regulated *Hbb-bh2* expression in *Nan* by quantitative RT-PCR. Average values for WT-PHB are shown in blue to facilitate comparison to *Nan*. AU, arbitrary units; *p < 0.01 and **< 0.001 *Nan* vs. WT-PHB. n = 5 (*Nan*) and 6 (WT-PHB) except Ortho (n = 2 and 3, respectively). (**g**) Adult β-globin, *Hbb-bs*, is downregulated in *Nan* pro- and basophilic erythroblasts. The duplicated adult globin locus, *Hbb-bt*, shows the same pattern (http://nanexpression.mdibl.org/). Neither *Hbb-bh2* nor *Hbb-bt* are differentially expressed in *Nan* whole fetal liver. Tick marks, mean ± SD. Note: All expression values are log2-transformed, normalized counts; therefore, values below 1 are considered background (no expression).

We showed previously that expression of *Hamp* and *Irf7* is significantly up-regulated in *Nan* whole fetal liver and adult whole spleen vs. WT fetal liver and spleen^15^, and confirmed upregulation in fetal liver here (Supplementary Table S2). Importantly, *Hamp* and *Irf7* upregulation was confirmed by qRT-PCR in fetal liver cells depleted of adherent cells and expanded in culture with erythropoietin in previous studies^15^. These data indicate that *Hamp* and *Irf7* are ectopically activated by Nan-KLF1 in fetal liver, as neither is normally expressed in erythroid cells^17,18^. Here, however, we find no *Hamp* expression in *Nan* or WT-PHB spleen erythroid precursors (http://nanexpression.mdibl.org/). Thus, *Hamp* expression driven by mutant Nan-KLF1 differs in fetal liver and anemic adult spleen erythroid cells even though both are stress environments. On the other hand, *Irf7* is expressed at equally high levels in both *Nan* and WT-PHB spleen (Fig. 2a), indicating that *Irf7* can also be upregulated as a general consequence of stress erythropoiesis in the spleen and is not specific to *Nan*.

Niche-specific differential expression of *Nan* β-like globin genes is also seen. Embryonic βh1 (*Hbb-bh1*) is significantly upregulated in *Nan* vs. WT fetal liver and non-anemic (hereafter WT) spleen^1,15^. Here however, no differential upregulation is seen in adult *Nan* vs. WT-PHB spleen precursors (Fig. 2b), indicating that increased expression in the spleen is due to stress erythropoiesis in response to anemia, as documented previously^25,26^. βh1 is expressed at low levels in proerythroblasts and basophilic erythroblasts where the embryonic globin repressor *Bcl11a* is highly expressed in both *Nan* and WT-PHB (Fig. 2c). *Bcl11a* expression falls in poly- and orthochromatophilic erythroblasts; the decrease is greater in *Nan* than WT-PHB, but their βh1 expression is equivalent in these precursors (Fig. 2b). We confirmed βh1 and *Bcl11a* expression patterns by qRT-PCR (Fig. 2d). Two additional major silencers of *Hbb-bh1 in vivo*, *Zbtb7a and Myb*^27,28^, are not differentially expressed at any stage (Supplemental Fig. 1).

Unexpectedly, βh2 (*Hbb-bh2*) is upregulated dramatically in *Nan* spleen – reaching 534-fold in orthochromatophilic erythroblasts; no or little expression is seen in WT-PHB spleen (Fig. 2e). This result was verified by qRT-PCR (Fig. 2f) confirming an altered effect of Nan-KLF1 at this locus. Comparing transcript read numbers, *Nan* βh2 comprises 0.34% and WT-PHB just 0.001% of total β-like globin in orthochromatophilic erythroblasts (Supplementary Table S8). No differential expression of βh2 is seen in fetal liver. Adult β-globin expression is delayed in *Nan* spleen compared to WT-PHB; β-globin is decreased at the pro- and basophilic erythroblast stages but recovers by the poly- and orthochromatophilic stages (Fig. 2g).

### Patterns of gene expression in *Nan*

**Figure 3:**
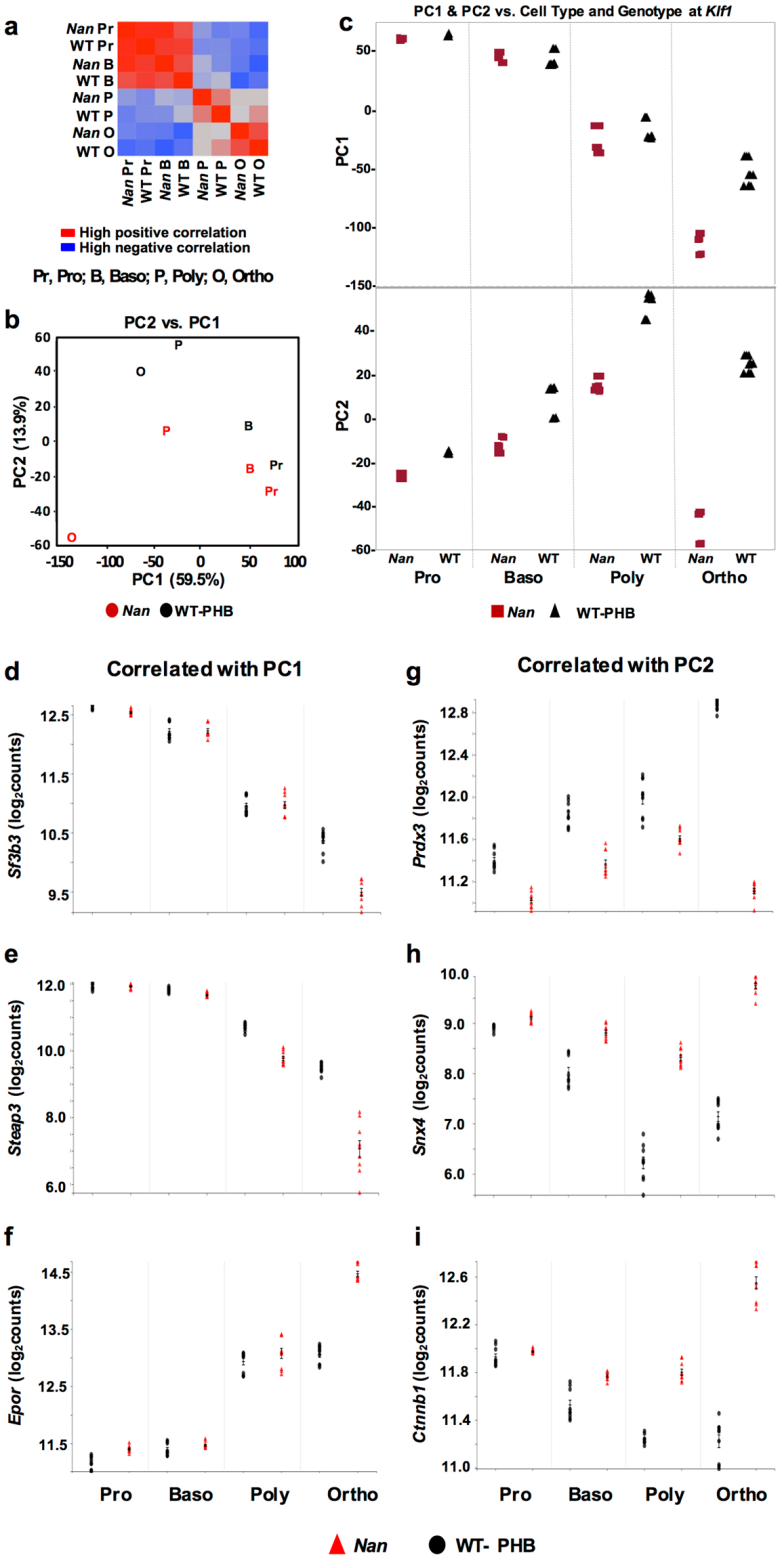
Principle component analysis (PCA). (**a**) Hierarchical clustering showing that expression differences are greatest in later stages of development (poly- and orthochromatophilic erythroblasts). (**b**) The first PC is dominated by cell type and accounts for 59.5% of the variation in expression while PC2 (13.9%) separates the samples within each cell type by genotype. (**c**) PC1 (top) and PC2 (bottom) plotted by genotype as differentiation proceeds from pro- orthochromatophilic erythroblasts reveal two major patterns of differential expression in *Nan*. (**d**) *Sf3b3* (splicing factor 3b, subunit 3) and (**e**) *Steap3* (STEAP family member 3) are positively, and (**f**) *Epor* (erythropoietin receptor) negatively correlated with PC1 where the most prominent expression differences are late in differentiation at the orthochromatophilic stage. (**g**) *Prdx3* (Peroxiredoxin 3) is negatively correlated with PC2 while (**h**) *Snx4* (Sorting nexin-4) and (**i**) *Ctnnb1* (β-catenin) are positively correlated with PC2. Genes correlated with PC2 show severe dysregulation throughout differentiation in *Nan* with a striking reversal at the orthochromatophilic stage. Tick marks, X ± SD.

We generated pairwise correlations of all samples using a reduced set (~8,600) of high variance genes. Hierarchical clustering based on these correlations shows that expression differences are much more severe in late (poly- and orthochromatophilic) than in early (pro- and basophilic) erythroblasts (Fig. 3a), confirming that progression of differentiation contributes a major source of expression variation. Indeed, PCA of the spleen expression matrix reveals that the first principle component (PC1) is dominated by cell type, whereas PC2 separates the samples within each cell type by genotype with modest effect of cell type at the pro- and basophilic stages but significantly more dramatic effects at the poly- and, particularly, the orthochromatophilic stages (Fig. 3b). PC1 and PC2 account for 59.5% and 13.9% of expression variation, respectively. The PCA confirms the role of cellular differentiation in driving expression variation as terminal differentiation proceeds with Nan-KLF1 driving increasingly aberrant transcription in later stages of differentiation.

When each PC is plotted separately as a function of genotype and precursor cell type, two major patterns of gene expression emerge in terms of how expression changes during differentiation from pro- to orthochromatophilic erythroblasts in WT-PHB precursors, and how expression diverges from WT-PHB in *Nan* precursors. Because PCA groups genes by their correlation with the components and represents both correlated and anti-correlated patterns, complimentary patterns of expression (meaning both increasing and decreasing) are included within each PC. Thus, the PC1 expression pattern is one of gradual, progressive change in expression in WT-PHB (either increasing or decreasing steadily) during differentiation, while expression in *Nan* shows a parallel overall course but progressively diverges from WT-PHB during differentiation such that expression differences are most prominent in poly- and orthochromatophilic erythroblasts (Fig. 3c, top panel). Examples of PC1-positively correlated genes (expression decreases during differentiation) include *Sf3b3* (splicing factor 3b, subunit 3) (Fig. 3d) and *Steap3* (STEAP family member 3) (Fig. 3e). *Epor* (erythropoietin receptor) exemplifies a PC1-negatively correlated gene (expression increases during differentiation) (Fig. 3f).

In the second expression pattern, that for PC2, expression in WT-PHB trends consistently up or down as differentiation proceeds (Fig. 3c, bottom panel). In *Nan*, expression is consistently higher or lower than WT-PHB at all stages and follows the same overall trend (either increasing or decreasing with differentiation) until the orthochromatophilic erythroblast stage, where a sharp, often dramatic reversal occurs in *Nan* (Fig. 3c, bottom panel). Moreover, while the direction of the differential expression in *Nan* is constant, the magnitude generally increases during differentiation. Thus, PC2-correlated genes show more severe dysregulation throughout differentiation in *Nan*. *Prdx3* (peroxiredoxin 3) is positively correlated with PC2 and its expression increases with differentiation; *Snx*4 (sorting nexin 4) and *Ctnnb1* (beta catenin) are negatively correlated (Fig. 3g–i) and expression decreases. The complete list of genes correlated with PC1 and PC2 is provided in Supplementary Table S9.

**Figure 4:**
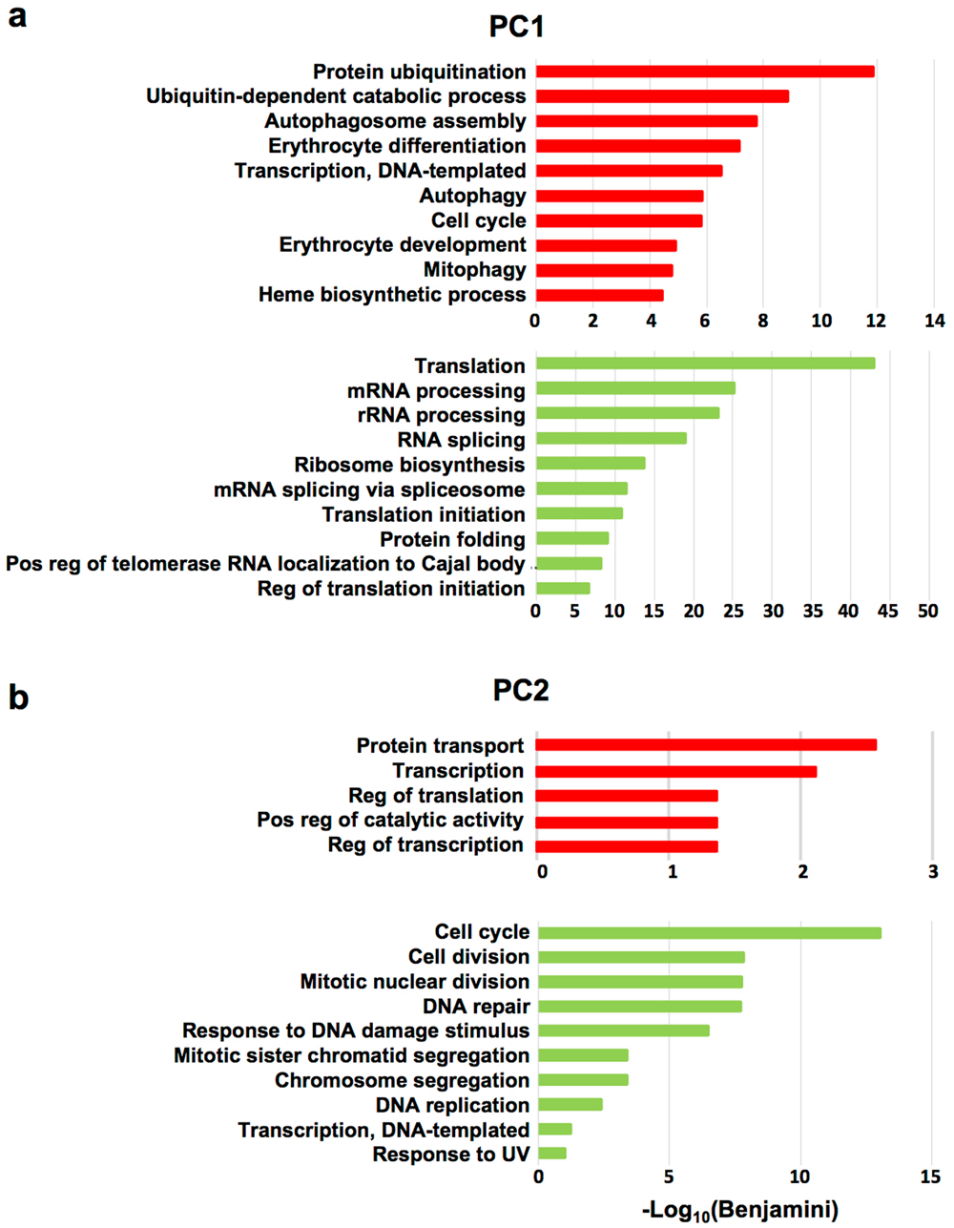
Functional annotation GO terms for PC1- and PC2- correlated DEGs. Top functional annotations associated with (**a**) PC1 and (**b**) PC2 DEGs. Genes negatively correlated with each PC are shown at the top in red and those positively correlated below in green. Note that correlation and expression are inversely related for PC1 genes (e.g., expression of negatively correlated genes increases during differentiation), but directly related in the case of PC2 (refer to Fig. 3c). Red and green also correspond to up- and down-regulated DEGs, respectively, in *Nan*. For this analysis, only the most significantly correlated genes (Supplemental Table 9) were included. For PC1, a cutoff of ±0.9 was used. For PC2, the cutoffs were −0.60 and +0.75 for negatively and positively correlated genes, respectively. Expanded correlation values were included for PC2 GO analysis due to the lower number of genes in this category.

We predict that the overall functional output of the altered *Nan* transcriptome will be most influenced by genes highly correlated with PC2, where *Nan* expression diverges the greatest throughout terminal differentiation, and to a lesser degree, PC1. For genes with the most significant negative correlations with PC1 (expression increases during differentiation), the top GO functional annotations include ubiquitination, autophagy/mitophagy, cell cycle, transcription, and erythroid differentiation (Fig. 4a). Conversely, the top annotations for the most significant PC1 positively correlated genes (expression decreases) are dominated by terms related to translation and mRNA processing. As PC1 is driven largely by cell type with little mutation effect, these up- and down changes in gene expression are consistent with major processes occurring during normal terminal differentiation. For PC2, where mutation effects dominate, protein transport, transcription and translation are the most significant GO functional annotations for positively correlated genes (expression increases); for negatively correlated genes (expression decreases) cell cycle, cell division, oxidation-reduction, and DNA repair dominate (Fig. 4b).

### Erythropoiesis in *Nan*

Previous studies revealed membrane skeleton defects and impaired globin expression in *Nan*. Here, based upon striking differential expression and predictions arising from PCA, we tested the hypothesis that additional processes go awry and contribute to anemia in *Nan*. We first examined erythropoiesis in *Nan* compared to both anemic and non-anemic WT spleen and bone marrow to achieve a full picture of erythroid status in *Nan*. Significantly, unlike KLF1-null mice^8^, expression of cell surface markers used to assess erythropoiesis by flow cytometry (Ter119, CD71, CD44) is normal or near normal in *Nan* (Supplemental Fig. 2).

**Figure 5:**
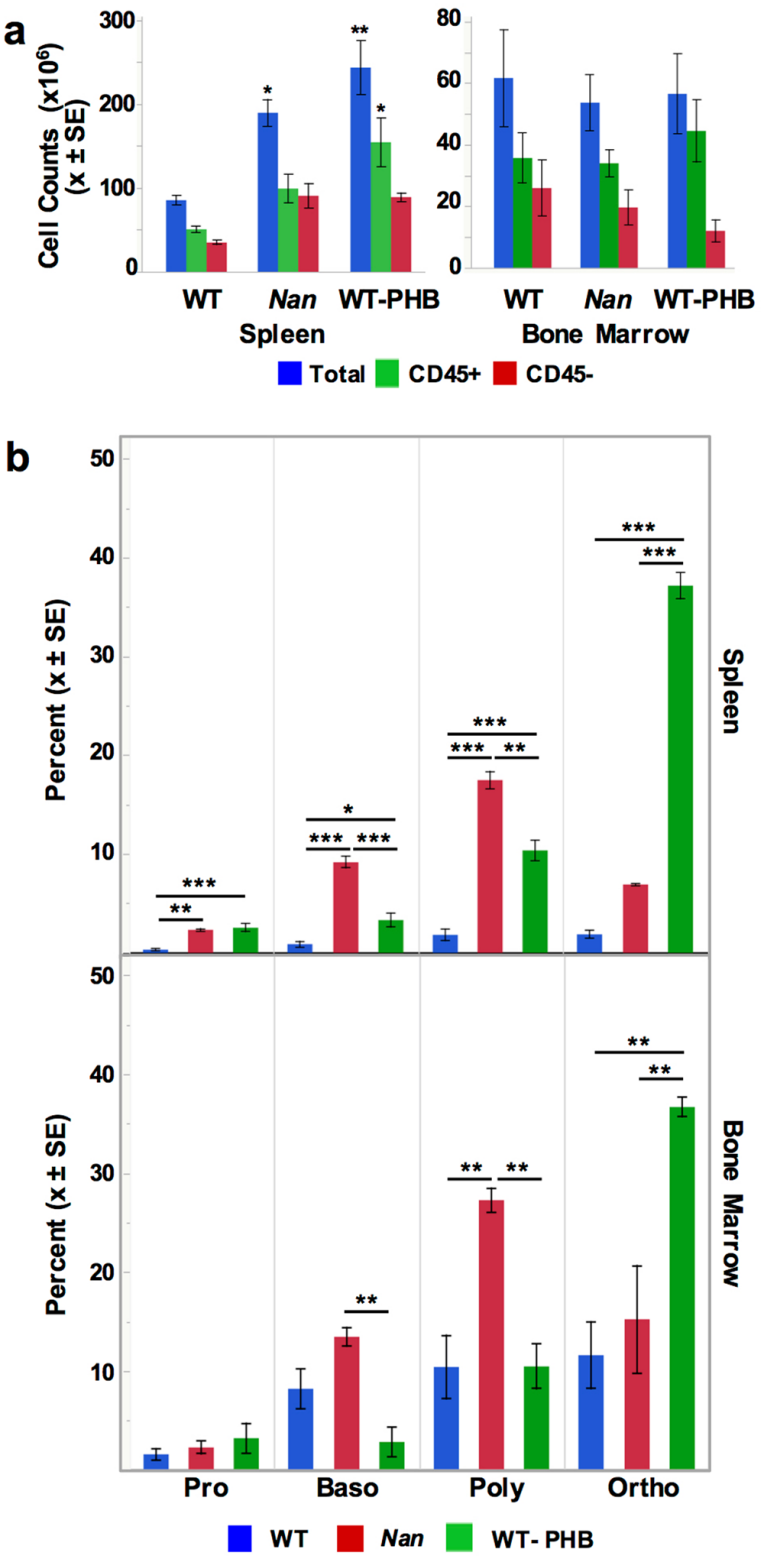
Erythropoiesis is delayed in *Nan* spleen. (**a**) Total, CD45 positive and CD45 negative cell counts in WT, *Nan*, and WT-PHB spleen (n = 3, 10, and 4, respectively) and bone marrow (n = 4/group). (**b**) Quantitation of the different spleen populations demonstrates an accumulation of pro-, baso and- polychromatophilic erythroblasts in *Nan* spleen (top panel) and baso- and- polychromatophilic erythroblasts in *Nan* bone marrow (bottom). As a result, production of orthochromatophilic erythroblasts markedly lags that of WT-PHB. n = 4/group. *P < 0.05; **p < 0.01; ***p < 0.001.

Spleen total cell counts (Fig. 5a) are significantly higher in *Nan* and WT-PHB vs. WT, as expected due to anemia. Non-erythroid (CD45+) cells are significantly increased in WT-PHB but not *Nan* compared to WT. Erythroid cell (CD45−) counts are increased approximately two-fold over WT in *Nan* and WT-PHB, but do not reach statistical significance. *Nan* erythroid cell counts showed more variability than WT and WT-PHB, overlapping WT in many cases (Supplemental Fig. 3), suggesting that stress erythropoiesis is inefficient to varying degrees in *Nan* mice. Bone marrow counts do not differ among the three groups (Fig. 5a).

Flow-cytometric analysis of differentiation^29^ revealed significantly delayed progression of erythropoiesis in *Nan* spleen, with cells accumulating at the proerythroblast stage compared to WT and at the baso- and polychromatophilic stages compared to both WT and WT-PHB (Fig. 5b). Orthochromatophilic erythroblasts are significantly increased in *Nan* compared to WT, but strikingly depleted compared to WT-PHB (Fig. 5b), as is also evident upon inspection of flow cytograms (Supplemental Fig. 4). The accumulation of earlier precursors with striking depletion of late orthochromatophilic erythroblasts in *Nan* vs. WT-PHB, where a progressive and dramatic increase in the percentages of each successive cell type is seen throughout differentiation, again suggests suboptimal stress erythropoiesis in *Nan*. A significant delay in erythropoiesis also occurs at the baso- and polychromatophilic stages in *Nan* bone marrow with depletion of orthochromatophilic erythroblasts compared to WT-PHB mice (Fig. 5b). These results confirm highly disordered basal and stress erythropoiesis *in Nan*.

### Cell cycle

**Figure 6:**
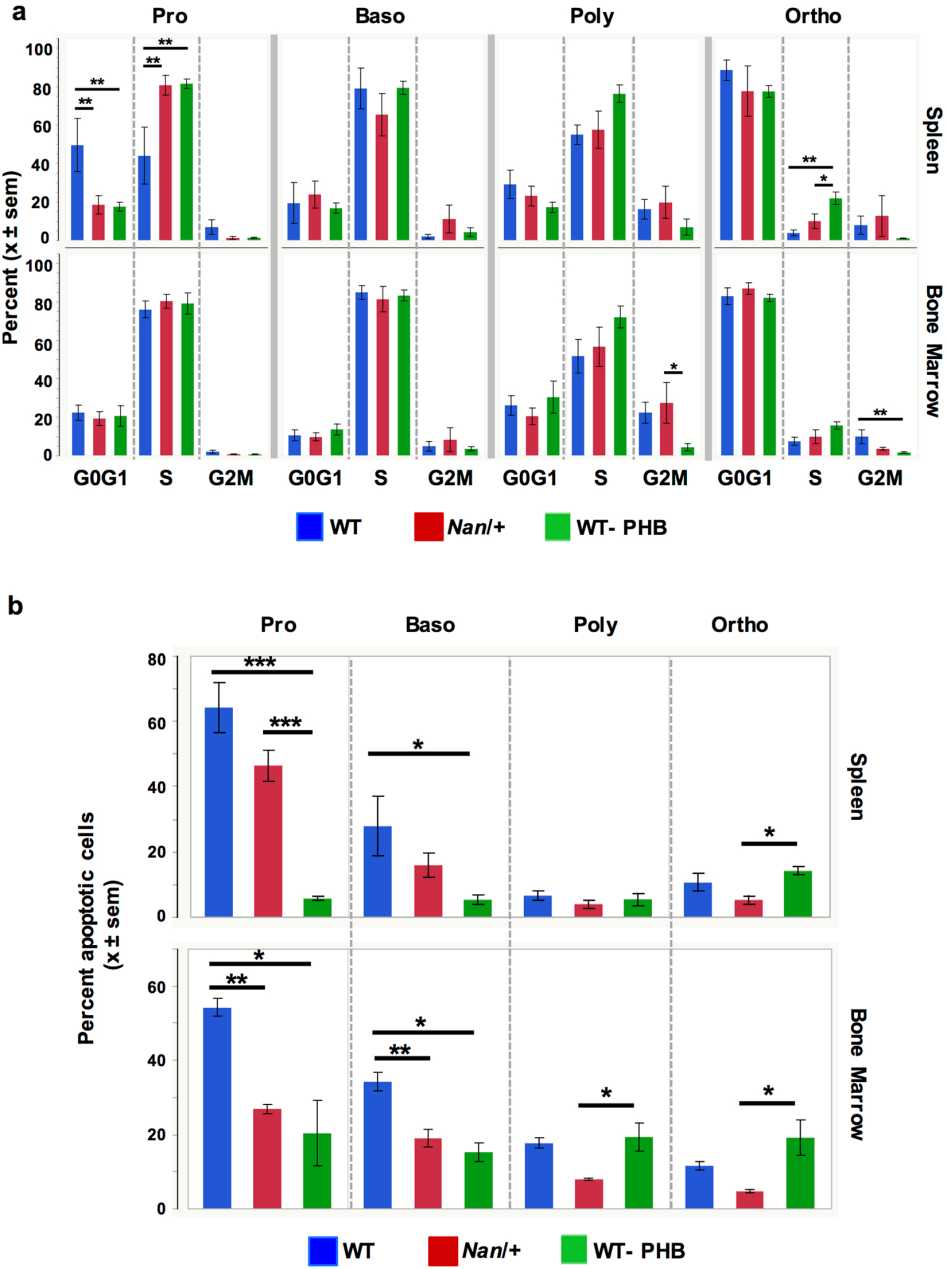
Cell cycle status and apoptosis in spleen and bone marrow. (**a**) Cell cycle status determined using BrdU in spleen (top) and bone marrow (bottom) erythroid precursors. Spleen, n = 6 (WT), 8 (*Nan*), 9 (WT-PHB); bone marrow, n = 3/group. (**b**) Apoptosis in spleen (top) and bone marrow (bottom) erythroid precursors. Spleen, n = 9 (WT), 10 (*Nan*), 7 (WT-PHB); bone marrow, n = 3 per group. *P < 0.05; **p < 0.01; ***p < 0.001.

We asked if cell cycle defects contribute to accumulation of precursors and delayed progression of maturation in *Nan*. Both *Nan* and WT-PHB spleen proerythroblasts, which do not differ from each other, show evidence of accelerated cell cycling compared to WT; the percentage of proerythroblasts in G0/G1 is significantly decreased and the percentage in S phase significantly increased (Fig. 6a). This pattern was not maintained, however, in subsequent precursors. In WT-PHB, no further differences except a small but significant increase in orthochromatophilic erythroblasts in S phase is seen. As expected, nearly all orthochromatophilic erythroblasts have exited the cell cycle. The percentage of *Nan* bone marrow polychromatophilic erythroblasts in G2M is significantly higher than WT-PHB but does not differ from WT (Fig. 6a). Thus, with the possible exception of spleen proerythroblasts, delayed differentiation with accumulation of precursors in *Nan* (Fig. 5) cannot be explained by cell cycle changes.

### Apoptosis

The percentage of apoptotic proerythroblasts in both WT and *Nan* spleen is dramatically and significantly higher than WT-PHB (Fig. 6b). Apoptotic basophilic erythroblasts are also higher in WT and *Nan vs*. WT-PHB, but do not reach statistical significance in *Nan*. Notably, apoptosis in *Nan* does not differ from WT in any spleen precursor population. Apoptosis in WT-PHB spleen is maintained at a low and consistent level throughout terminal differentiation in the spleen, while early precursors in *Nan* undergo significant apoptosis. Thus, a high percentage of pro-and basophilic erythroblasts are lost in the *Nan* spleen when increased RBC production is needed.

In bone marrow, apoptotic *Nan* and WT-PHB pro- and basophilic erythroblasts do not differ from each other but are significantly less than WT (Fig. 6b). Apoptosis in *Nan* poly- and orthochromatophilic erythroblasts is decreased relative WT.

### ROS generation, mitophagy

**Figure 7:**
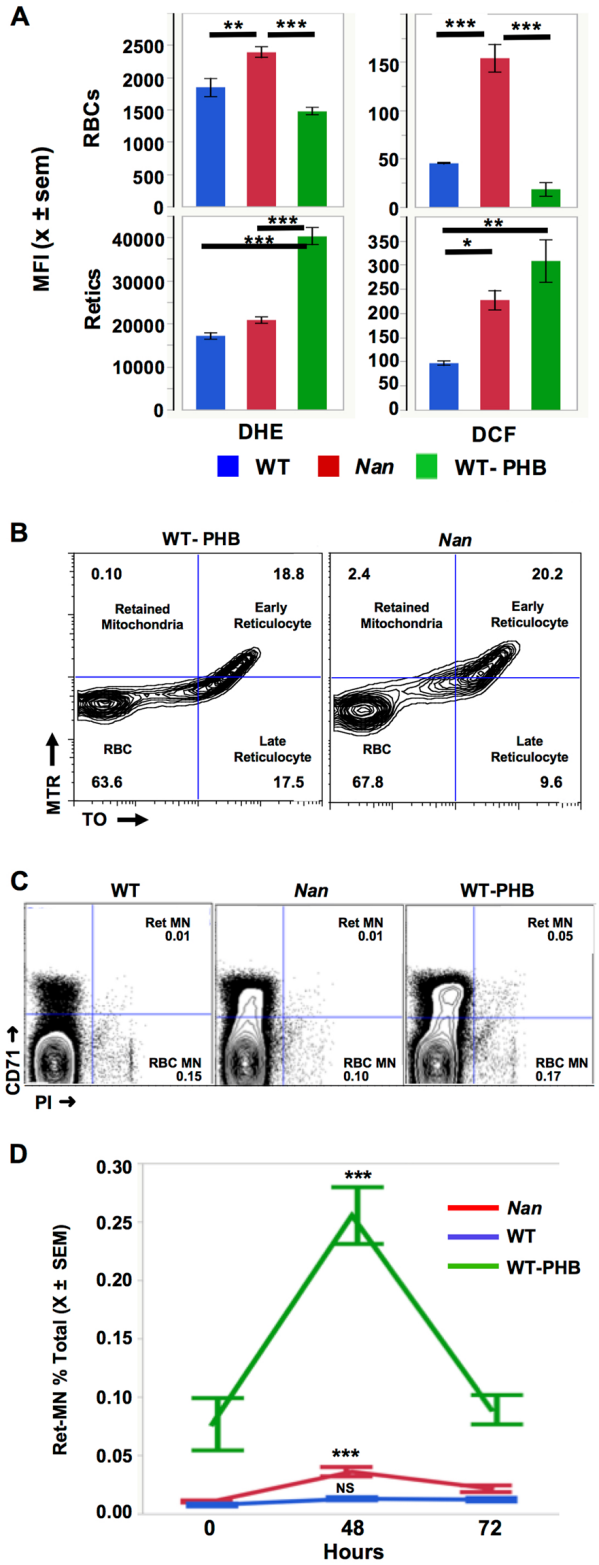
ROS generation, mitophagy, and DNA damage in *Nan*. (**A**) Quantitation of ROS production in Ter119 positive, CD71 low (mature red blood cells) and Ter119 positive CD71 high (reticulocytes) populations. As expected, DCF and DHE production in mature red cells is lower than in reticulocytes^43^. n = 3 per group. (**B**) Flow cytometry of MitoTracker Red (MTR) and Thiazole Orange (TO) stained WT and *Nan* red blood cells to assess mitochondria and ribosome content three days post-phlebotomy. Early reticulocytes are ribosome-positive, mitochondria-positive (upper right quadrant), late reticulocytes ribosome-positive, mitochondria-negative (lower right quadrant), and retained mitochondria ribosome-negative, mitochondria- positive (upper left quadrant). Cytograms representative of 3 samples/group. (**C**) Representative micronucleus assay results in non-irradiated mice. (**D**) Quantitation of induced micronuclei 48 and 72 hours following irradiation. n = 4 per group; ***p < 0.001 (within group comparison at 0 and 48 hours). PI, propidium iodide.

To measure ROS, we utilized peroxide- and superoxide-sensitive fluorescent dyes, DCF and DHE, respectively. Preliminary studies suggested increased ROS generation in *Nan* whole blood*;* subsequent analyses were done on CD71 and Ter119 stained whole blood to obtain quantitative data on enriched reticulocyte (CD71^hi^) and mature erythrocyte (CD71^lo^) fractions (Supplemental Fig. 5). Superoxide and peroxide generation is increased in *Nan* mature erythrocytes compared to WT and WT-PHB (Fig. 7A). In *Nan* reticulocytes peroxide generation did not differ significantly from WT but was significantly less than WT-PHB. Reticulocyte superoxide generation in *Nan* and WT-PHB did not differ but both were increased compared to WT (Fig. 7A).

Increased ROS production can result from increased mitochondrial content due to defective mitophagy. Hence, we examined RBC mitochondria and ribosome content using MitoTracker Red and Thiazole Orange (Fig. 7B). A small increase in retained mitochondria is seen in *Nan* compared to WT (1.5 ± 0.8 vs. 0.2 ± 0.2, X ±SD, n = 3) although the difference was not statistically significant. Thus, increased ROS production without significant mitochondria retention contributes to hemolysis in *Nan*.

### DNA damage

We examined DNA damage using a flow-cytometric peripheral blood micronucleus (MN) assay, which provides a quantitative measure of both spontaneous and radiation induced chromosome damage^30,31^. A small, but significant, increase in *Nan* reticulocyte MN is seen 48 hours post-irradiation (Fig. 7C,D), returning to baseline (time 0) at 72 hours. A similar pattern, although exaggerated compared to *Nan*, is seen in WT-PHB. The exaggerated response in WT-PHB may be due to acute induction of anemia in this group. Overall, these data indicate that WT-PHB and *Nan* are more susceptible to induced MN formation vs. WT; however, the DNA damage response is intact, as reticulocyte MN returned to baseline at 72 hours in both. RBC MN, indicative of spontaneous damage, did not change pre-and post-irradiation, as expected (not shown).

## Discussion

Prior studies revealed that Nan-KLF1 acts as both a hypomorphic and neomorphic allele, failing to bind a subset of normal KLF1 target genes while inappropriately engaging non-KLF1 targets^13^. The result is a corrupted transcriptome that produces anemia by both cell intrinsic and extrinsic mechanisms^15^. *Nan* is relevant to human disease where a mutation at the corresponding position causes CDA type IV; the different mouse-human anemia phenotype may reflect the different amino acid changes.

Here, we compared adult *Nan* and WT-PHB sorted spleen erythroid precursors as a way to discern effects on gene expression specific to Nan-KLF1 induced anemia. We chose phlebotomy-induced anemia over phenylhydrazine treatment to avoid confounding factors induced by phenylhydrazine such as widespread oxidative damage, severe hemolysis, and near 100% reticulocytosis. RNAseq revealed progressively increasing transcriptome changes in *Nan* during erythroid differentiation. PCA revealed that PC1 was dominated by cell type, contributing 60% of the expression variation. PC2, dominated by genotype effects, contributed 14% with strongest effects in poly- and orthochromatophilic cells reflecting increasing mutant *Klf1* expression during differentiation. In total, 6,965 unique genes were differentially expressed with only a subset overlapping those identified in fetal liver.

We assessed overall functional effects of the extensive global transcriptome changes in the context of the whole animal. We first examined erythropoiesis by flow cytometry and found evidence of suboptimal stress erythropoiesis in *Nan*. Increased percentages of all erythroid precursors are seen in WT-PHB compared to WT spleen, as expected during stress erythropoiesis in response to anemia. *Nan*, however, shows a significant accumulation of pro-, baso-, and polychromatophilic erythroblasts compared to WT, and a significant accumulation of baso- and polychromatophilic erythroblasts in conjunction with a severe decrement of orthochromatophilic erythroblasts compared to WT-PHB. These data suggest that progression though the final stages of differentiation fails in a significant portion of *Nan* precursors, results concordant with analyses of KLF1-null fetal liver erythroblasts^32^. Strikingly, erythropoiesis in the *Nan* bone marrow shows the same overall pattern where a significant accumulation of polychromatophilic over orthochromatophilic cells is also evident. Despite the decrement in orthochromatophilic erythroblasts in *Nan*, reticulocytosis is significantly elevated, possibly due to slower maturation or faster kinetics in reaching the peripheral circulation over WT, or both. Whether the latter occurs at the level of enucleation and/or extrusion from the bone marrow and spleen remains to be elucidated. Nonetheless, total circulating RBCs (reticulocytes plus mature erythrocytes) are significantly decreased in *Nan*.

Two major patterns of expression divergence between WT-PHB and *Nan* during differentiation emerged when each PC was plotted individually as a function of cell type and genotype (Fig. 3c). Compared to PC1, PC2 show relatively greater divergence of expression in *Nan* precursors, particularly late precursors. Using top functional annotation GO terms associated with PC2 as a starting point, we identified processes including cell cycle, DNA damage, and apoptosis that could contribute to anemia with delayed progression of erythropoiesis and/or suboptimal stress erythropoiesis in *Nan* for functional screening. In terms of the cell cycle, significant changes indicative of increased cell cycle progression were detected in *Nan* and WT-PHB proerythroblasts compared to WT but were not maintained in later precursors. Overall, cell cycle changes fail to account for disordered erythropoiesis in *Nan*. Likewise, although *Nan* appears more susceptible to induced DNA damage, the DNA damage response is intact.

Interestingly, differences in apoptosis could at least partially underlie suboptimal stress erythropoiesis in *Nan*. Previous studies^33^ demonstrated that stress-responsive pro- and basophilic erythroblasts undergo a high rate of apoptosis in non-anemic WT mice, and that this response is suppressed in anemic mice under the influence of erythropoietin (Epo). Here, greater than 60% of WT spleen proerythroblasts and nearly 30% of basophilic erythroblasts are apoptotic; spleen apoptosis in early WT-PHB precursors, most strikingly proerythroblasts, is markedly decreased compared to WT (Fig. 6b). In *Nan*, despite anemia accompanied by high serum erythropoietin (Epo) levels^15^ and normal *Epor* expression in stress-responsive precursors (Fig. 3i; nanexpression.mdibl.org), apoptosis is inappropriately high, not differing significantly from WT. Epo-responsive mechanisms mediating suppression of apoptosis in anemia and facilitating erythroid expansion during stress in WT mice include downregulation of cell-surface expression of apoptosis-promoting Fas and FasL, and induction of anti-apoptotic Bcl-x_L_^33–35^. *Bcl2l1* encoding Bcl-x_L_ is also a well described direct KLF1 target gene^36^. KLF1 and STAT5 likely direct expression of *Bcl2l1* via independent intronic enhancers^35^. Neither Fas nor FasL are expressed in WT-PHB or *Nan* early erythroblasts and, therefore, cannot account for the observed differences in apoptosis. Similarly, *Bcl2l1* is not differentially expressed in *Nan* pro- and basophilic erythroblasts, although expression is decreased slightly (nanexpression.mdibl.org). Nonetheless, unless decreased *Bcl2l1* expression in *Nan* to levels considerably below our criteria for statistical significance (absolute fold change >2, p < 0.05) affects apoptosis, Bcl-x_L_ seems unlikely to mediate failure to downregulate apoptosis in early *Nan* precursors. In general, inappropriate up-regulation of pro-apoptotic genes in *Nan*, or inappropriate down-regulation of anti-apoptotic genes, could account for failure to suppress apoptosis. Of the many other anti-apoptotic genes in addition to *Bcl2l1* that are expressed in erythroid precursors (*Bcl2*, *Mcl1*, *Bcl2l2*, *Bcl2L10*), none are downregulated in *Nan* pro- and basophilic erythroblasts. Likewise, none of the major pro-apoptotic genes^37–39^ expressed in erythroid precursors including *Bax*, *Bak1*, *Bok*, *Bid*, *Bcl211* (BIM), *Bbc3* (PUMA), *Bad*, *Pmaip1* (NOXA) *or Hrk* are significantly upregulated in *Nan*. Thus, although apoptosis clearly plays a role in stress erythropoiesis, what drives the different apoptotic rates in *Nan* vs. WT-PHB spleen precursors is presently unknown. Elucidating mechanisms underlying the inadequate stress response in *Nan* will require additional study.

ROS is generated in RBCs primarily through the autooxidation of oxyhemoglobin, which produces superoxide ions^40^. Increased ROS generation is seen in *Nan* and WT-PHB peripheral blood cells, although the major species and relative amounts produced differ in *Nan* vs. WT-PHB reticulocytes and mature red blood cells (Fig. 7A). Increased ROS causes or exacerbates anemia via oxidative damage to membranes causing hemolysis and decreased erythrocyte lifespan. To combat the deleterious effects of ROS, red cells rely on superoxide dismutases to convert superoxide to hydrogen peroxide, which is then scavenged by peroxiredoxins, catalase, and glutathione peroxidase^40–42^. Of the major antioxidants at work in the red cell, the peroxiredoxins (*Prdx1*/*2*) and superoxide dismutase (*Sod2*) are well characterized in terms of their null phenotypes leading to increased RBC ROS generation and hemolytic anemia in mice^41–44^. In *Nan*, expression of *Prdx1* and *Prdx2* is erythroid precursors is decreased, but not significantly. Notably, *Prdx3*, *4*, 5, and 6 are robustly expressed in anemic erythroid precursors. *Prdx5* is not differentially expressed, but expression of both *Prdx3* and 4 is decreased 3.4 fold in *Nan* orthochromatophilic erythroblasts, and expression of *Prdx6* is increased in both poly- (2.8- fold) and orthochromatophilic (2.5-fold) erythroblasts. Mice null for *Prdx3* and 4 are associated with increased oxidative stress in adipose^45^ and testis^46^, respectively, but a role for these peroxiredoxins, or for *Prdx5* and 6, in erythrocytes has not previously been noted to our knowledge. We hypothesize that these peroxiredoxins, like *Prdx1* and 2, play a role in oxidant defense in RBCs, and that their dysregulated expression may contribute to anema in *Nan* Similarly, four glutathione peroxidase genes, *Gpx1*, 3, 4, and 7 are expressed in anemic spleen, and all but *Gpx1* are dysregulated in *Nan*. Thus, these and other proteins involved in red cell oxidant defense could contribute as well.

Interestingly, functional annotations relevant to ribosomes and translation dominate PC1 genes whose expression decreases with differentiation. Indeed, an extensive list of, large (*Rpl*−) and small (*Rps*−) ribosomal subunit genes are differentially expressed, mostly downregulated, in *Nan* (Supplementary Table S10). Despite this, no evidence of ribosomopathy is seen. Haploinsufficiency of twenty ribosomal subunit genes is known or suspected to cause the inherited bone marrow failure syndrome, Diamond Blackfan anemia (DBA)^47^, and all of but two of these genes are downregulated in *Nan*. For example, *Rps19*, the cause of 25% of DBA cases, is decreased >six-fold. However, *Nan* bone marrow is normocellular without any decrement in erythroid precursors^47–50^, peripheral RBCs are microcytic, and reticulocyte production is elevated, all inconsistent with DBA or other bone failure syndromes.

Despite large fold changes in many genes associated with specific cellular processes, such as translation, function is maintained. Large numbers of GO terms associated with cell cycle, as another example, are found in both the up-and down-regulated gene lists in all precursors (Supplementary Table S11), but significant detectable consequences in *Nan* are few. Thus, tolerance to expression variation is high, at least in the context of the red cell. Possibly, absolute expression remains sufficient to maintain function. Also, expression changes that are the direct consequence of Nan-KLF1 would be expected to cause indirect downstream effects on other genes; these could compensate for Nan-KLF1 induced changes and allow the most critical basic cellular functions to remain intact, leading to physiological resiliency in developing red cells *in vivo*. Future studies will be needed to determine the extent to which expression tolerance is reflected at the posttranscriptional level.

## Methods

### Animals

All mice were maintained at The Jackson Laboratory in climate- and light cycle-controlled rooms. Acidified water and 5K52 chow (PMI LabDiet) were provided *ad libitum*. *Nan* is a fully inbred strain. The original mutant *Nan* mouse was a female offspring of an ENU–mutagenized C3H/101 F1 male and a “PT” female at the Medical Research Council, Harwell, U.K. The PT stock was a mixed-background mutation-testing stock. The anemic female was mated to an untreated C3H/101 F1 male, and the mutation was propagated by intercrossing offspring^1,2^. *Nan* was imported to the Jackson Laboratory in 1994 and maintained in the research colony of Dr. Luanne Peters by brother-sister matings for more than 20 generations. Hence, according to mouse nomenclature rules (http://www.informatics.jax.org/mgihome/nomen/), the original Harwell *Nan* stock used in this study is a fully inbred subline designated NAN/Llp. The official designations for homozygous, heterozygotes and WT littermates are NAN/Llp *Klf1*^*Nan*^/*Klf1*^*Nan*^, NAN/Llp *Klf1*^*Nan*^/Klf1^+^, and NAN/Llp *Klf1*^+^/*Klf1*^+^, respectively. All experiments were carried out in accordance with National Institutes of Health Laboratory Animal Care Guidelines and were approved by the Animal Care and Use Committee (ACUC) of the Jackson Laboratory.

### Phlebotomy

Adult WT littermates were phlebotomized via daily retro-orbital collection of ~350 μL whole blood followed immediately by intraperitoneal injection of sterile normal saline (37 °C) for four days.

### Peripheral blood studies

Complete blood counts were determined using a Siemens Advia 120 analyzer. Blood smears were stained with Wright-Giemsa (Sigma-Aldrich). Serum lactate dehydrogenase (LDH) and bilirubin levels were determined using a Beckman AU680 analyzer.

### Sorting of erythroid precursors

Single cell suspensions of adult (6–8 weeks) whole spleens were depleted of CD45+ cells using mouse CD45 MicroBeads (Miltenyi Biotec), stained (CD45-FITC, Ter119-PE and CD44-APC), and sorted on a FACSAria II flow cytometer (BD Biosciences) as described^29^.

### RNAseq

Total RNA was extracted with the PureLink RNA Mini Kit (Invitrogen) and libraries prepared using the KAPA mRNA HyperPrep Kit (KAPA Biosystems), according to the manufacturer’s instructions. Briefly, the protocol entails isolation of polyA containing mRNA using oligo-dT magnetic beads, RNA fragmentation, first and second strand cDNA synthesis, ligation of Illumina-specific adapters containing a unique barcode sequence for each library, and PCR amplification. Libraries were checked for quality and concentration using the D5000 ScreenTape assay (Agilent Technologies) and quantitative PCR (KAPA Biosystems), according to the manufacturer’s nstructions. Paired end sequencing was performed on the HiSeq2500 System using TruSeq SBS Kit v4 reagents (Illumina). Reads were aligned to mouse genome GRCm38 with STAR Aligner^51^. Quality control was performed with RSeQC^52,53^ and Picard (http://broadinstitute.github.io/picard/). Quantification to the mouse transcriptome ENSEMBL v85 was implemented using Gencode version M10^54^. Count matrix normalization (rlog transformation) and differential expression analysis was performed with DESeq2^55^. Differential expression analysis was performed on unnormalized counts assigned to each gene rather than FPKM (Fragments Per Kilobase of gene per Million reads), as recommended for DESeq2. Differentially expressed genes were extracted from DESeq2 output files using threshold values of ≥2 absolute fold change and ≤0.05 adjusted p-value.

Previously obtained fetal liver RNAseq data^13^ (SRA SRP061646, GEO GSE71396) was re-analyzed as above to facilitate valid comparisons between datasets.

To examine global patterns, further analysis was performed on the rlog regularized matrix.

The regularized matrix was further Z-transformed by the function *Z* = (*exp* − *avg*)/*stdev*, where *avg* and *stdev* are the average and sample standard deviation of the regularized expression level of each gene across all samples. To focus the analysis on genes with appreciable expression and/or variation, the matrix was reduced from all 45,232 annotated genes to 8,668 with *avg* ≥ 5.0 or *stdev* ≥ 2.0. Principal Components Analysis was performed using default parameters with JMP v11.2.1 (SAS Institute) on the transpose of the reduced Z-transformed expression matrix. Gene ontology (GO) term searches were performed with DAVID v6.8 (default settings)^24^.

### Sanger sequencing

cDNA was synthesized using the Ambion MessageSensor RT kit and Invitrogen random decamer primers (Invitrogen). PCR was performed using Invitrogen Platinum SuperFi PCR Master Mix (Invitrogen) with an annealing temperature of 65 °C. *Klf1* forward (5′-AGACGCAGGCTTGTCCCCGGGAACT) and reverse (5′-GCAGGATCACTCAGAGGTGACGCTT) primers were in exons 2 and 3, respectively, and spanned the *Nan* mutation. PCR products are purified using MagBIO’s HighPrep PCR magnetic beads. Sequencing was performed using Applied Biosystems BigDye Terminator ready reaction kit Version 3.1, run on an Applied Biosystems 3730xl, and analyzed using Applied Biosystems Sequencing Analysis software version 5.2.

### Quantitative real-time RT-PCR (qRT-PCR)

cDNA was prepared as above. qRT‐PCR reactions consisted of 4.5 μl of cDNA, 5 μl of TaqMan Gene Expression Master Mix and 0.5 μl of primers and probes (TaqMan® gene expression assay, assay ID: *Hbb-bh1*, Mm00433932_g1; *Hbb-bh2*, Mm01273444_g1; *Bcl11a*, Mm00479358_m1) (Applied Biosystems). Samples were prepared in triplicate. qRT-PCR was performed using a ViiA7 Real-Time PCR System (Applied Biosystems) (40 cycles at 95 °C for 15 sec and 60 °C for 1 min). The relative quantification of each gene was determined by the 2^−∆∆CT^ method using control CT values and *Gapdh* (TaqMan® gene expression assay ID: Mn99999915_g1) expression for normalization.

### Erythropoiesis, cell cycle and apoptosis

Terminal erythroid differentiation was monitored using CD44, Ter119, and forward scatter as described^29^ and analyzed using a LSRFortessa and FlowJo v9.9.4 software. Cell cycle and apoptosis were analyzed using BD Bioscience’s APC BrdU Flow Kit and Annexin V^56^, respectively, per manufacturer’s instructions.

### ROS production, mitophagy and DNA damage

Intracellular ROS accumulation was determined using chloromethyl, dichlorodihydrofluorescein diacetate (M-H2DCFDA) and dihydroethidium (DHE) (ThermoFisher) as described^43^. Samples were analyzed on a FACSymphony A5 flow cytometer (BD Biosciences) and FlowJo v9.9.4. Mitophagy was assessed using Mitotracker Red CMXRos (MTR; Molecular Probes) and thiazole orange (TO, BD Biosciences) as described^57^. DNA damage was assessed by micronucleus assay at baseline and following irradiation as previously described^30,31^. Micronuclei (MN) arise from chromosomes or chromosome fragments that are not incorporated into the nucleus during cell division and remain in peripheral blood cells. As reticulocytes arise following the last mitosis, radiation induced chromosome damage is detected as an increase in reticulocyte MN. Intrinsic or spontaneous damage is seen as an increase in MN in mature red cells, which do not contain a nucleus at the time of irradiation.

### Data Acquisition and Imaging

Images of peripheral blood were acquired using a Nikon Eclipse E600 microscope equipped with a Nikon DS-Fi3 color camera and NIS-Elements Documentation imaging software (Nikon). Smears were examined with a 100/0.5–1.3 oil-immersion objective.

### Statistics

Significant differences were identified using Tukey honestly significant differences (HSD) or 2-tailed Student *t* test using JMP Statistical Software v11.2.1.

### Data Deposition

RNAseq data were submitted to Sequence Read Archive (Bioproject PRJNA432205). Expression profiles are available at http://nanexpression.mdibl.org/).

## Electronic supplementary material

Supplementary Tables

Supplementary Figures
