# Supplementary material for: Mutant KLF1 in Adult Anemic Nan Mice Leads to Profound Transcriptome Changes and Disordered Erythropoiesis
Source: Sci Rep. 2018 Aug 24;8:12793. doi: 10.1038/s41598-018-30839-2 (PMC6109071; doi:10.1038/s41598-018-30839-2)
Supplement: Supplementary file 2 — Supplementary Figures [file 41598_2018_30839_MOESM2_ESM.pdf]

Mutant KLF1 in Adult Anemic *Nan* Mice Leads to Profound Transcriptome Changes and Disordered Erythropoiesis

Danitza Nébor, Joel H. Graber, Steven L. Ciciotte, Raymond F. Robledo, Julien Papoin, Emily Hartman, Kevin R. Gillinder, Andrew C. Perkins,
James J. Bieker, Lionel Blanc, and Luanne L. Peters

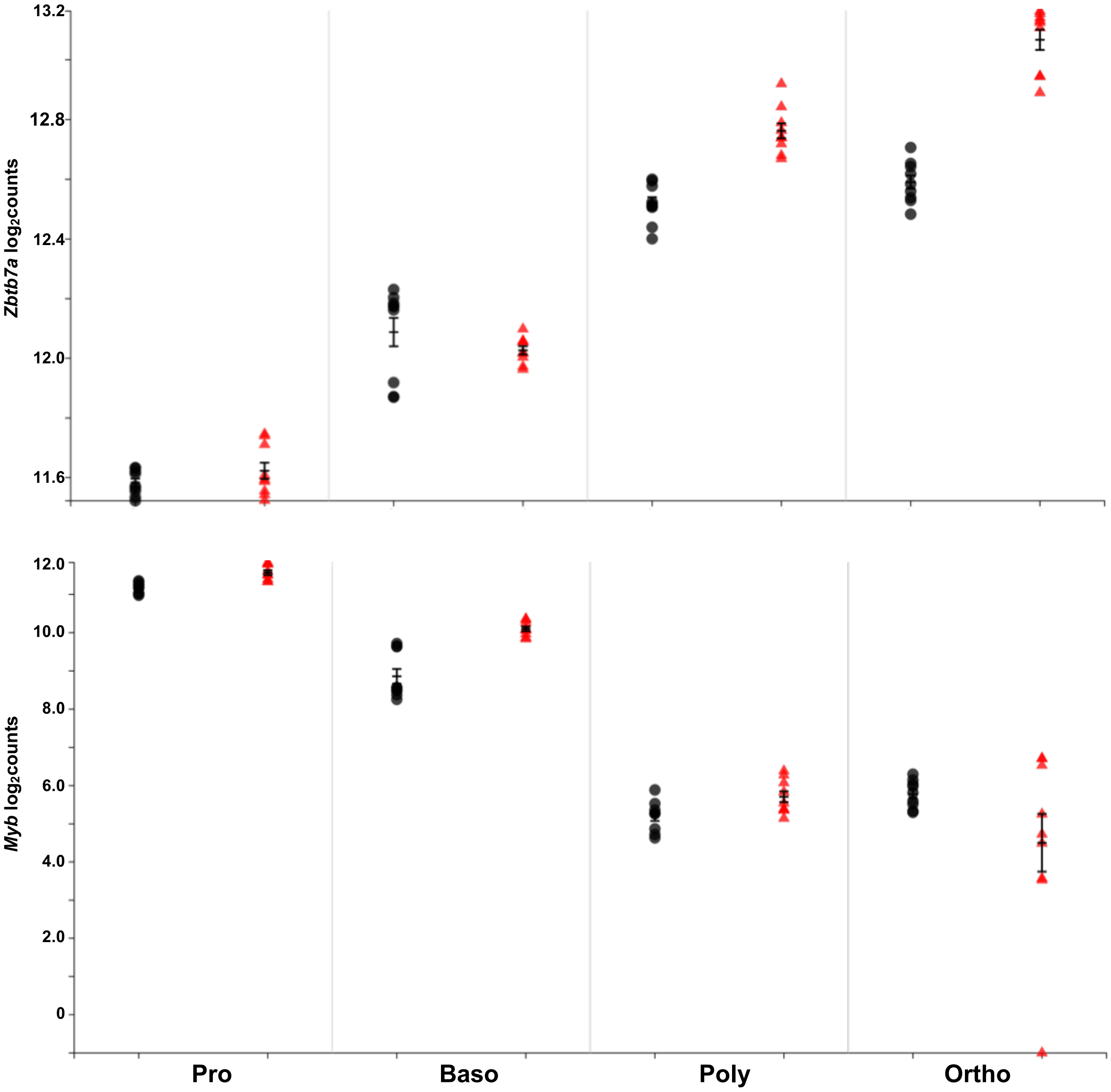

Supplemental figure 1. Expression of *Zbtb7a* and *Myb* in *Nan* and WT-PHB spleen erythroid precursors (RNAseq).

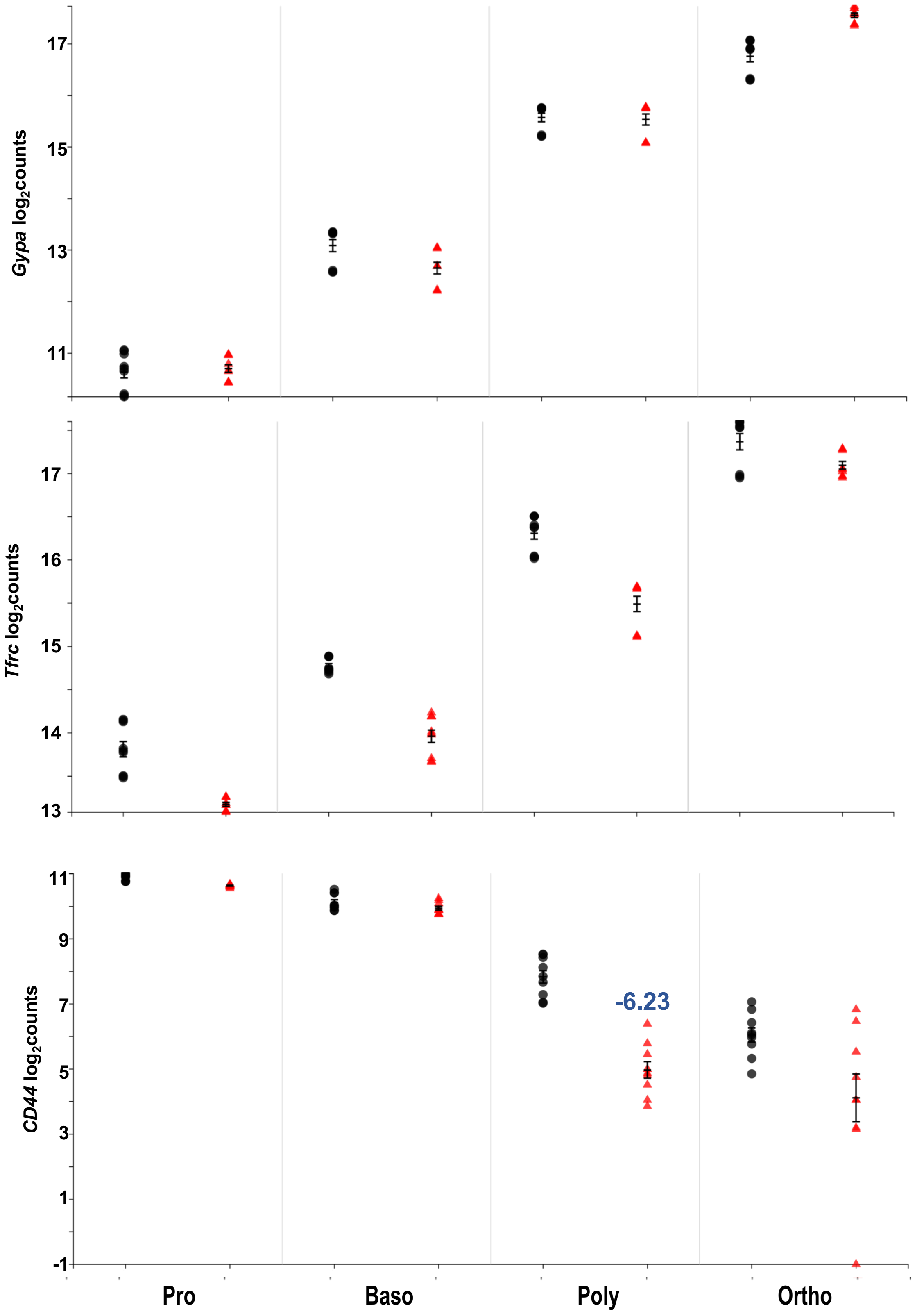

Supplemental figure 2. Surface markers for flow-cytometric analysis of erythropoiesis. With the exception of CD44 in polychromatophilic erythroblasts, which is downregulated in *Nan* (6.23 fold), no significant differential expression is seen in *Nan* vs. WT-PHB precursors (see also supplemental Tables 3-6). *Gypa* encodes mouse Ter119; *Tfrc* encodes surface marker CD71 (transferrin receptor).

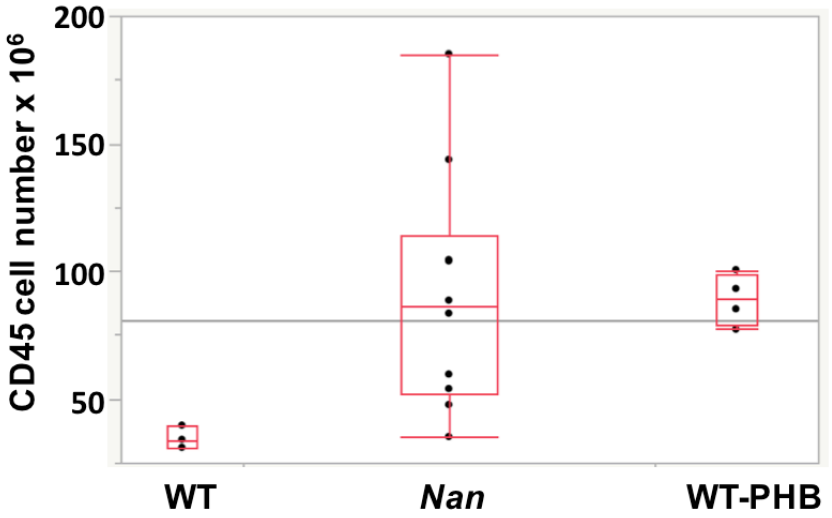

Supplemental figure 3. Boxplots illustrating range of values for erythroid cell counts in WT, *Nan* and WT-PHB spleen.

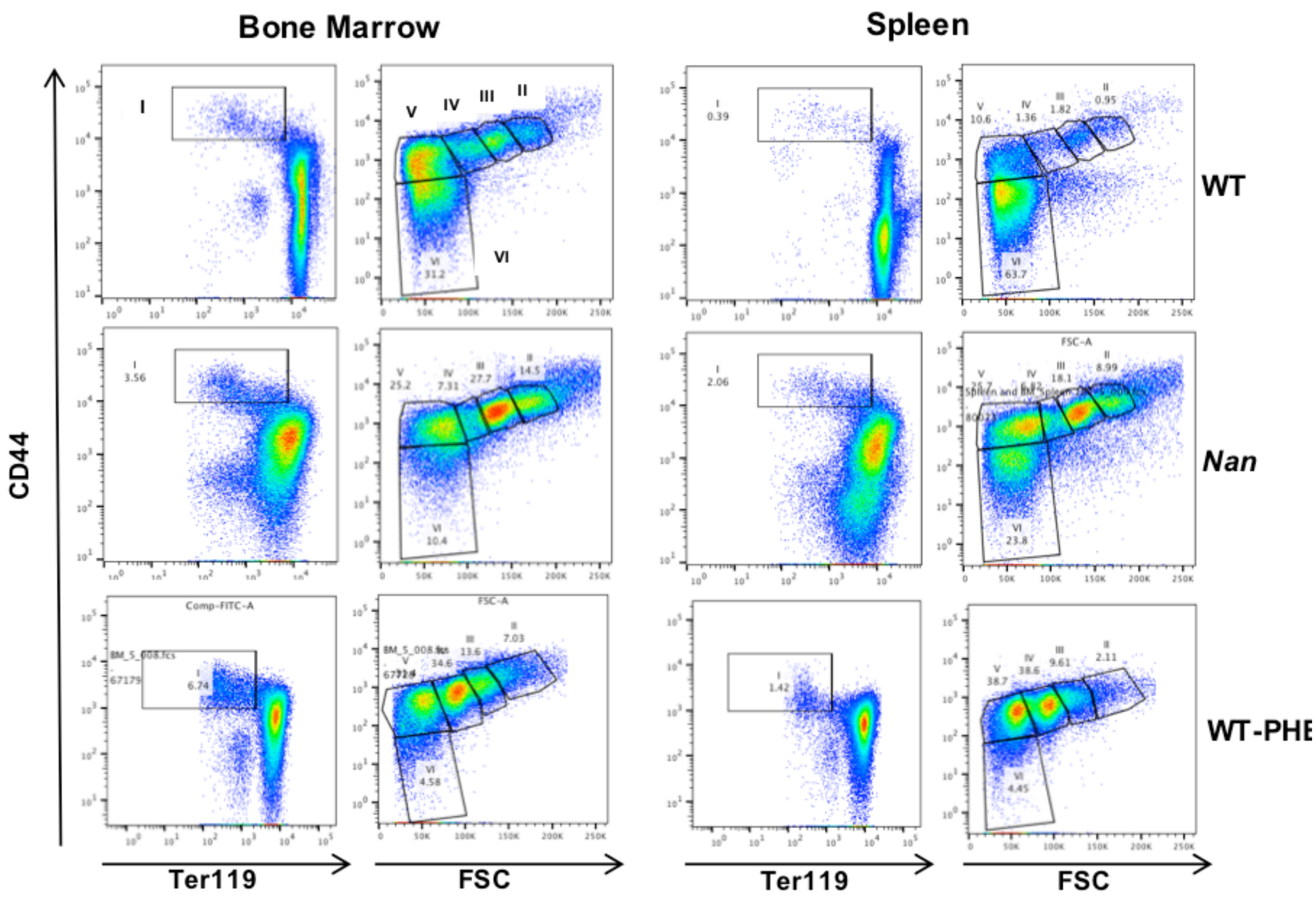

Supplemental figure 4. Flow cytograms showing gating of cell populations in bone marrow and spleen. I, pro-; II, baso-; III, Poly-; IV, orthochromatophilic erythroblasts; V, reticulocytes; VI, mature red cells

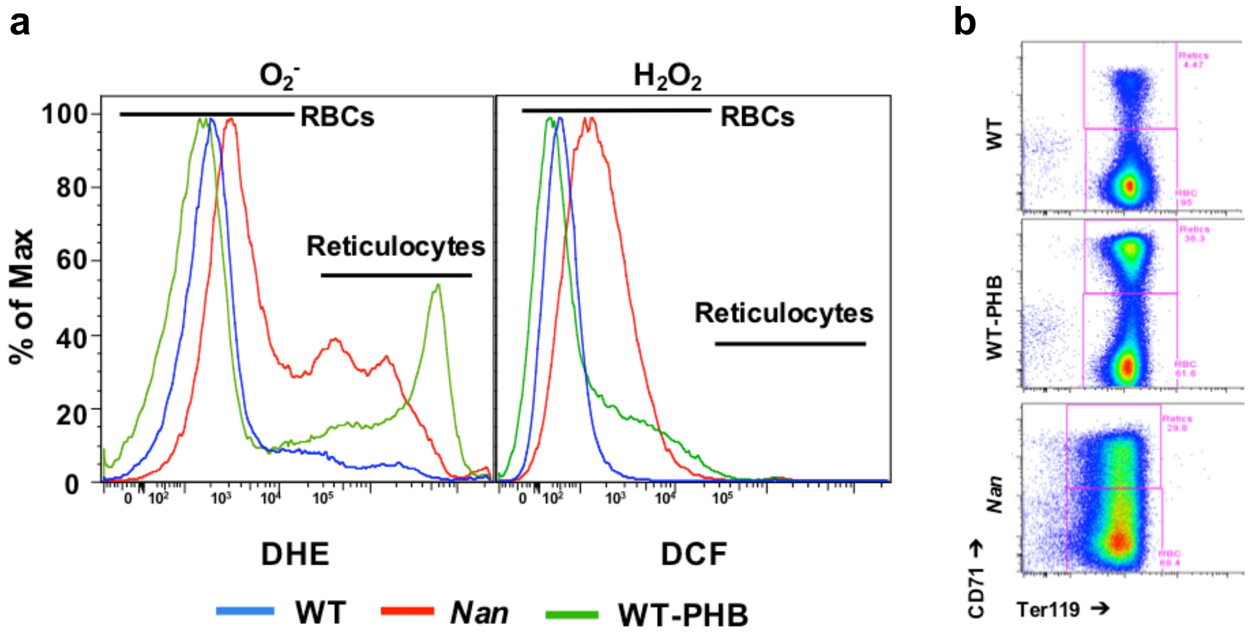

Supplemental figure 5. (a) Representative flow cytometric analysis of WT, WT-PHB, and *Nan* whole blood incubated with superoxide-sensitive dye DHE and peroxide-sensitive H2DCFDA (DCF). Mature red cells are the major peak while reticulocytes, which produce higher ROS levels, are in the tail. **(b)** Separation of reticulocyte and RBCs populations in whole blood using CD71 and Ter119.
